# Evaluation of Targeted Next-Generation Sequencing for the Management of Patients Diagnosed with a Cancer of Unknown Primary

**DOI:** 10.1093/oncolo/oyab014

**Published:** 2022-01-28

**Authors:** Michael J Fusco, Todd C Knepper, Juliana Balliu, Alex Del Cueto, Jose M Laborde, Sharjeel M Hooda, Andrew S Brohl, Marilyn M Bui, J Kevin Hicks

**Affiliations:** 1 Department of Individualized Cancer Management, Section for Precision Oncology, Moffitt Comprehensive Cancer Center, Tampa, FL, USA; 2 Department of Biostatistics and Bioinformatics, Moffitt Comprehensive Cancer Center, Tampa, FL, USA; 3 Department of Satellite and Community Oncology, Moffitt Comprehensive Cancer Center, Tampa, FL, USA; 4 Sarcoma Department, Moffitt Comprehensive Cancer Center, Tampa, FL, USA; 5 Department of Pathology, Moffitt Comprehensive Cancer Center, Tampa, FL, USA

**Keywords:** next generation sequencing, cancer genetics, cancer of unknown primary, pharmacogenetics, precision medicine

## Abstract

**Background:**

Cancer of unknown primary (CUP) comprises a heterogeneous collection of malignancies that are typically associated with a poor prognosis and a lack of effective treatment options. We retrospectively evaluated the clinical utility of targeted next-generation sequencing (NGS) among CUP patients to assist with diagnosis and identify opportunities for molecularly guided therapy.

**Patients and Methods:**

Patients with a CUP at Moffitt Cancer Center who underwent NGS between January 1, 2014 and December 31, 2019, were eligible for study inclusion. Next-generation sequencing results were assessed to determine the frequency of clinically actionable molecular alterations, and chart reviews were performed to ascertain the number of patients receiving molecularly guided therapy.

**Results:**

Ninety-five CUP patients were identified for analysis. Next-generation sequencing testing identified options for molecularly guided therapy for 55% (*n* = 52) of patients. Among patients with molecularly guided therapy options, 33% (*n* = 17) were prescribed a molecularly guided therapy. The median overall survival for those receiving molecularly guided therapy was 23.6 months. Among the evaluable patients, the median duration of treatment for CUP patients (*n* = 7) receiving molecular-guided therapy as a first-line therapy was 39 weeks. The median duration of treatment for CUP patients (*n* = 8) treated with molecularly guided therapy in the second- or later-line setting was 13 weeks. Next-generation sequencing results were found to be suggestive of a likely primary tumor type for 15% (*n* = 14) of patients.

**Conclusion:**

Next-generation sequencing results enabled the identification of treatment options in a majority of patients and assisted with the identification of a likely primary tumor type in a clinically meaningful subset of patients.

## Teaser

Cancers of unknown primary are associated with poor prognosis and few effective treatment options. This article evaluates the clinical utility of targeted next-generation sequencing among patients with cancers of unknown primary.

**Implications for Practice**

The majority of patients with a cancer of unknown primary have a historically poor prognosis. Data have suggested that next-generation sequencing (NGS) could assist with identifying targeted therapy options for cancer of unknown primary patients that is inclusive of checkpoint immunotherapy. In the present study, favorable treatment outcomes were observed in patients treated based upon NGS results when compared with historical data for unknown primary cancer patients. For a subset of patients, NGS results assisted with identification of likely primary tumor type.

## Introduction

Cancer of unknown primary (CUP) is by definition a histologically confirmed metastatic cancer for which clinicians are unable to identify a primary tumor after a standard diagnostic approach.^1^ The American Cancer Society has estimated that over 30 000 individuals will be diagnosed with a CUP in 2021, which is approximately 2% of all new cancer diagnoses.^2^ Cancer of unknown primary is typically treated with chemotherapeutic agents that have been shown to have activity across a broad spectrum of malignancies.^3^ Regimens utilizing a doublet containing either taxane or platinum agents are commonly used, but meta-analyses have reported modest responses to most chemotherapy regimens with median overall survival ranging from 3 to 10 months.^3-6^

The clinical implementation of precision oncology has advanced in recent years, driven in part by an increasing number of commercially available drugs targeting specific genomic alterations. In the last 3 years alone, there have been over 25 new FDA approvals for anticancer drugs that have biomarker eligibility criteria included in their labeled indication.^7^ As the number of molecularly guided anti-cancer therapies continues to grow, there will be increased opportunities for off-label targeted therapy for CUP patients found to harbor targetable genomic alterations.^8,9^ Furthermore, approval of molecularly guided anticancer therapies that are tumor type-agnostic are emerging. Both entrectinib and larotrectinib are approved for neurotrophic tyrosine receptor kinase (NTRK) fusion-positive solid tumors,^10,11^ independent of tumor type, and pembrolizumab is approved for solid tumors with microsatellite instability (MSI-High) as well as those with a high tumor mutation burden (TMB-High).^12^ Basket and umbrella trials such as the NCI MATCH trial and the TAPUR trial are also available to patients with solid tumors harboring specific genomic alterations.

The European Society for Medical Oncology (ESMO) and the National Comprehensive Cancer Network (NCCN) have both published recommendations regarding the utilization of next-generation sequencing (NGS) among patients with CUP.^3^ The ESMO Precision Medicine Working Group recently published recommendations for the use of NGS among patients diagnosed with metastatic cancers, with large panel NGS recommended as a consideration for those with a CUP diagnosis.^13^ National Comprehensive Cancer Network guidelines recommend that NGS testing can be considered based on clinicopathologic features in situations where it would guide therapeutic decision-making in patients with localized adenocarcinoma or carcinoma not-otherwise-specified as a way to identify potentially actionable genomic aberrations. National Comprehensive Cancer Network guidelines also highlight that, “until more robust outcomes and comparative effectiveness data are available, pathologists and oncologists must collaborate on the judicious use of immunohistochemistry, gene expression profiling, and NGS on a case-by-case basis.” We acknowledge that there is a need for additional insight regarding the clinical integration of somatic tumor profiling to advance the individualized treatment of this heterogeneous patient subset. The purpose of this study was to evaluate the clinical utility of NGS to guide treatment decisions in a cohort of patients with unknown primary cancer in a real-world setting.

## Materials and Methods

### Study Population

This retrospective study was conducted at H. Lee Moffitt Cancer Center and Research Institute in accordance with an Institutional Review Board-approved protocol. The protocol was approved by Moffitt Cancer Center in accordance with the Declaration of Helsinki and the 21st Century Cures Act. Patients aged 18 years and older with a CUP diagnosis were eligible for inclusion. Eligible patients were identified using a clinical database that discretely stores abstracted NGS results along with patient demographics inclusive of diagnosis.^14^ The database was searched for patients with a CUP diagnosis who had an NGS assay performed between January 1, 2014, and December 31, 2019. A comprehensive chart review was completed for each eligible patient utilizing the electronic health record (EHR). The clinical course for each patient was determined including lines of therapy received, duration of treatment, and overall survival.

### Somatic NGS Testing

Next-generation sequencing testing was performed utilizing commercially available platforms including FoundationOne, FoundationOne Heme, FoundationOne ACT, FoundationOne CDx, and Guardant360 along with an in-house NGS assay referred to as Moffitt STAR. These platforms have been described in depth elsewhere previously.^15-17^ Choice of the assay was at the discretion of the treating physician.

### Evaluation of Next-Generation Sequencing Results

Clinically actionable alterations identified by NGS were classified based upon the Precision Oncology Knowledge Base (OncoKB) classification version 2 using the levels of evidence that are pertinent to CUPs.^18^ Levels of evidence are defined as follows: (1) level 1 genomic alterations are those FDA-recognized biomarkers predictive of response to an approved drug inclusive of CUP diagnosis, (2) level 3B genomic alterations are those predictive of response to an FDA-approved drug in another cancer type, (3) level 4 genomic alterations are those with compelling biological evidence predictive of response to a drug. By definition, the OncoKB levels 2 and 3A categories for the level of evidence are currently not pertinent to CUPs due to a lack of a known tumor type. An additional level 3C was created for genomic alterations predictive of response to investigational agents only. This distinction was made to more clearly quantitate eligibility for off-label drug therapy (level 3B) and opportunities for clinical trials (level 3C) eligible to solid tumors inclusive of CUP. The genomic alteration with the highest level of evidence for molecularly guided treatment was documented for each patient. Patients were categorized as having favorable or unfavorable prognoses based on NCCN and ESMO guidelines.^3,5^

For the purpose of this study, molecularly guided therapeutic opportunities were categorized as either targeted therapy or checkpoint immunotherapy eligible. Targeted therapy eligibility was defined as the opportunity to use an anticancer drug that targets a genetic mutation predicted to be oncogenic. Immunotherapy eligibility was defined as those with MSI or TMB-High. Tumor mutational burden was classified as TMB-Low (<10 mutations/megabase [Mb]) or TMB-High (≥10 mutations/Mb) based upon the tumor agnostic FDA-approval of pemborlizumab for the treatment of unresectable or metastatic TMB-High (≥10 mutations/Mb) solid tumors.^12,19^ Additionally, homologous recombination repair genes included in the indication for olaparib use in prostate cancer were assessed as level 3B alterations in CUP.

Based upon prior work by Tothill et al,^20^ in which molecular features of tumors were utilized as indicators of a likely tissue of origin, we also sought to evaluate the utility of NGS in assisting with diagnosis of a likely tumor type based upon the probability of a mutation or combination of mutations being present in a given tumor type. To determine whether molecular features assisted with diagnosis, an EHR chart review was performed to determine whether the primary oncologist referenced the NGS results as supportive of a likely primary tumor type. From EHR chart review, it was also ascertained whether patients received genetic counseling based upon findings from somatic NGS testing. Genetic results for those referred to genetic counseling and underwent germline testing were documented.

### Outcomes

Treatment outcomes were assessed for patients who received molecularly guided treatment options, including overall survival, treatment duration, and Von Hoff ratio. Overall survival was measured from the date of pathologic diagnosis to the date of death. As this was a retrospective study, treatment duration was used as a surrogate marker of progression-free survival. The Von Hoff method was utilized to evaluate the clinical benefit of molecularly guided therapy in patients with cancer of unknown primary. The Von Hoff method utilizes each patient as their own control by comparing progression-free survival (PFS2, defined as time on therapy) of the molecularly guided therapy to the progression-free survival (PFS1) for the therapy administered immediately prior (PFS2/PFS1).^21-23^ A ratio of PFS2/PFS1 ≥1.3 is proposed to be a surrogate marker of clinical benefit.^21-23^ The Von Hoff method was chosen as an endpoint for its ability to assess clinical utility in a heterogeneous population.

### Statistical Analysis

Median overall survival was calculated for treatment groups defined as molecularly guided therapy and non-molecularly guided therapy (hereafter referred to as “standard options”). The Kaplan–Meier method was used to produce survival curves. The log-rank method was used to test for the difference between survival rates in each treatment group. Hazard ratios and 95% confidence intervals were calculated with the use of univariate Cox proportional hazards, with treatment as a covariate.

## Results

### Patients with CUP

Ninety-five patients were eligible for inclusion in this study with a median age of 65 years old (range: 18 to 85) at diagnosis of CUP and a median age of 68 years at the time of NGS. Fifty-two percent of patients were female, with most patients (93%) self-declaring race as white (Table 1). Adenocarcinoma (43%) was the most prevalent histology followed by carcinoma not otherwise specified (25%). Patients had a median of 1 prior line of therapy (range: 0 to 8) before undergoing NGS. Fourteen patients (15%) were categorized as having a favorable prognosis including those with poorly differentiated neuroendocrine carcinoma, squamous cell carcinoma involving non-supraclavicular cervical lymph nodes, or diagnosed with a CUP having a colorectal signature based on immunohistochemistry and molecular profiling.

**Table 1.** Patient demographic and clinical characteristics.

Patient characteristics	Molecularly guided therapy group (*n* = 17)	Standard treatment options group (*n* = 78)	Entire cohort (*N* = 95)
Age at sequencing years, ­median (range)	59 (18 to 92)	69 (24 to 83)	68 (18 to 92)
Prior lines of therapy, ­median (range)	1 (0 to 8)	1 (0 to 6)	1 (0 to 8)
Sub-group			
Favorable prognosis	18% (*n* = 3)	14% (*n* = 11)	15% (*n* = 14)
Unfavorable prognosis	82% (*n* = 14)	86% (*n* = 67)	85% (*n* = 81)
Sex			
Female	53% (*n* = 9)	51% (*n* = 40)	52% (*n* = 49)
Male	47% (*n* = 8)	49% (*n* = 38)	48% (*n* = 46)
Race (self-declared)			
White	100% (*n* = 17)	91% (*n* = 71)	93% (*n* = 88)
Black	0% (*n* = 0)	5% (*n* = 4)	4% (*n* = 4)
Asian	0% (*n* = 0)	1% (*n* = 1)	1% (*n* = 1)
Other	0% (*n* = 0)	3% (*n* = 2)	2% (*n* = 2)
Smoking status			
Current smoker	18% (*n* = 3)	10% (*n* = 8)	12% (*n* = 11)
Former smoker	29% (*n* = 5)	56% (*n* = 44)	52% (*n* = 49)
Never smoker	53% (*n* = 9)	33% (*n* = 26)	37% (*n* = 35)
Histology			
Adenocarcinoma	24% (*n* = 4)	47% (*n* = 37)	43% (*n* = 41)
Carcinoma NOS	53% (*n* = 9)	19% (*n* = 15)	25% (*n* = 24)
Squamous cell	12% (*n* = 2)	12% (*n* = 9)	12% (*n* = 11)
Carcinoma			
Neuroendocrine	6% (*n* = 1)	8% (*n* = 6)	7% (*n* = 7)
Adenosquamous	6% (*n* = 1)	1% (*n* = 1)	2% (*n* = 2)
Undifferentiated	0% (*n* = 0)	1% (*n* = 1)	1% (*n* = 1)
Other	0% (*n* = 0)	12% (*n* = 9)	10% (*n* = 9)

Abbreviation: NOS, not otherwise specified.

### Therapeutic Options Identified

**Figure 1.**
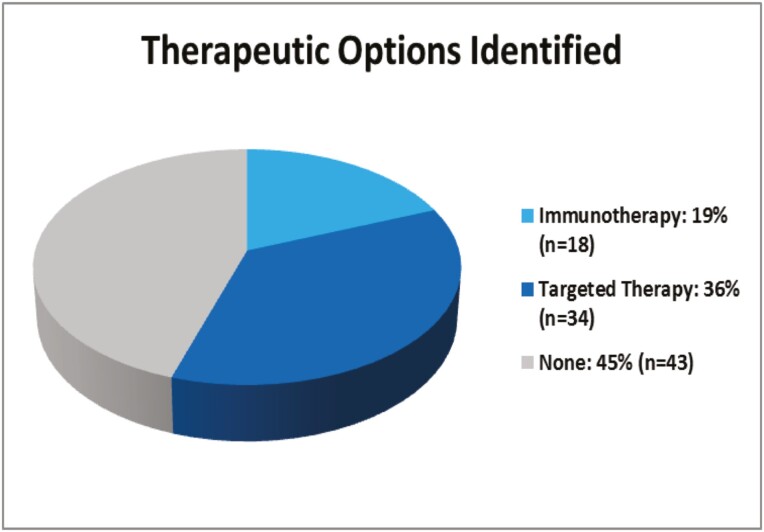
Therapeutic opportunities identified utilizing next-generation sequencing to guide the treatment of cancer of unknown primary.

There were a total of 68 clinically actionable alterations observed among 52 (55%) patients with cancer of unknown primary (Table 2). Therapeutic options identified included eligibility for checkpoint immunotherapy (*n* = 18) and targeted therapy (*n* = 34) (Figure 1). This included 18 (19%) patients with NGS results revealing opportunities for on-label therapy (level 1 evidence), 30 (32%) with level 3B evidence for off-label therapy, and 4 (4%) with level 3C evidence for investigational therapies as the highest-level of evidence identified. Thirteen (14%) patients had more than one clinically actionable alteration (level 3 evidence or higher).

**Table 2.** Levels of evidence for biomarkers identified with NGS.

Level of evidence^a^	% (*n*)^b^
Level 1	
MSI-High^c^	3% (3)
TMB-High^d^	16% (15)
Level 3B	
*ALK* Fusion	1% (1)
*ATM*	4% (4)
*BRAF* V600E	2% (2)
*BRCA1*	4% (4)
*BRCA2*	2% (2)
*CHEK2*	2% (2)
*ERBB2* (Amp 2, Mut 4)	6% (6)
*FGFR2* Fusion	2% (2)
*IDH1*	4% (4)
*IDH2*	1% (1)
*MET* (Amp 1)	1% (1)
*NRAS*	1% (1)
*PIK3CA*	7% (7)
*PALB2*	1% (1)
*PTCH1*	3% (3)
*RET*	1% (1)
Level 3C	
*HRAS*	1% (1)
*KRAS* G12C	7% (7)
Level 4	
*ATR* (1)	1% (1)
*BRAF* Non-V600E	2% (2)
*KRAS* Non-G12C	21% (20)
*NF1*	6% (6)
*PTEN*	5% (5)
None	23% (22)

Level of evidence based on FDA-approved drugs and available clinical trials at the time of data analysis. Drugs such as sotorasib that targets KRAS G12C were approved after data analysis along with the availability of additional clinical trials (eg, telaglenastat for NF1-mutated tumors).

Individual patients may have a CUP harboring more than 1 targetable mutation.

All MSI-High tumors also had a high tumor mutational burden.

Includes only microsatellite stable (MSS) tumors.

Taking all genetic variants into consideration, 18 patients were found to be MSI-High (*n* = 3) or TMB-High (*n* = 15) (Table 2). Off-label therapeutic opportunities included *BRAF* V600E mutations (*n* = 2), *ERBB2* amplification or mutation (*n* = 2 and *n* = 4, respectively), mutations in *IDH1* or *IDH2* (*n* = 4 and *n* = 1, respectively), *MET* amplification (*n* = 1), and mutations in *NRAS* (*n* = 1), *PIK3CA* (*n* = 7), *PTCH1* (*n* = 3), or *RET* (*n* = 1). Tumor suppressor genes implicated in homologous recombination repair deficiency (HRD) were a recurrent feature of the cohort, including *ATM* (*n* = 4), *BRCA1* (*n* = 4), *BRCA2* (*n* = 2), *CHEK2* (*n* = 2), and *PALB2* (*n* = 1) (Table 2). Oncogenic fusions were identified in three CUP patients, including *ALK* (*n* = 1) and *FGFR2* rearrangements (*n* = 2). Eight patients were eligible for clinical trial opportunities based on the presence of an HRAS mutation (*n* = 1) or *KRAS* G12C mutations (*n* = 7). Of note, sotorasib was recently approved for locally advanced or metastatic *KRAS* G12C-mutated non-small-cell lung cancer patients who have received at least one prior systemic therapy.^24^

### Outcomes

**Figure 2.**
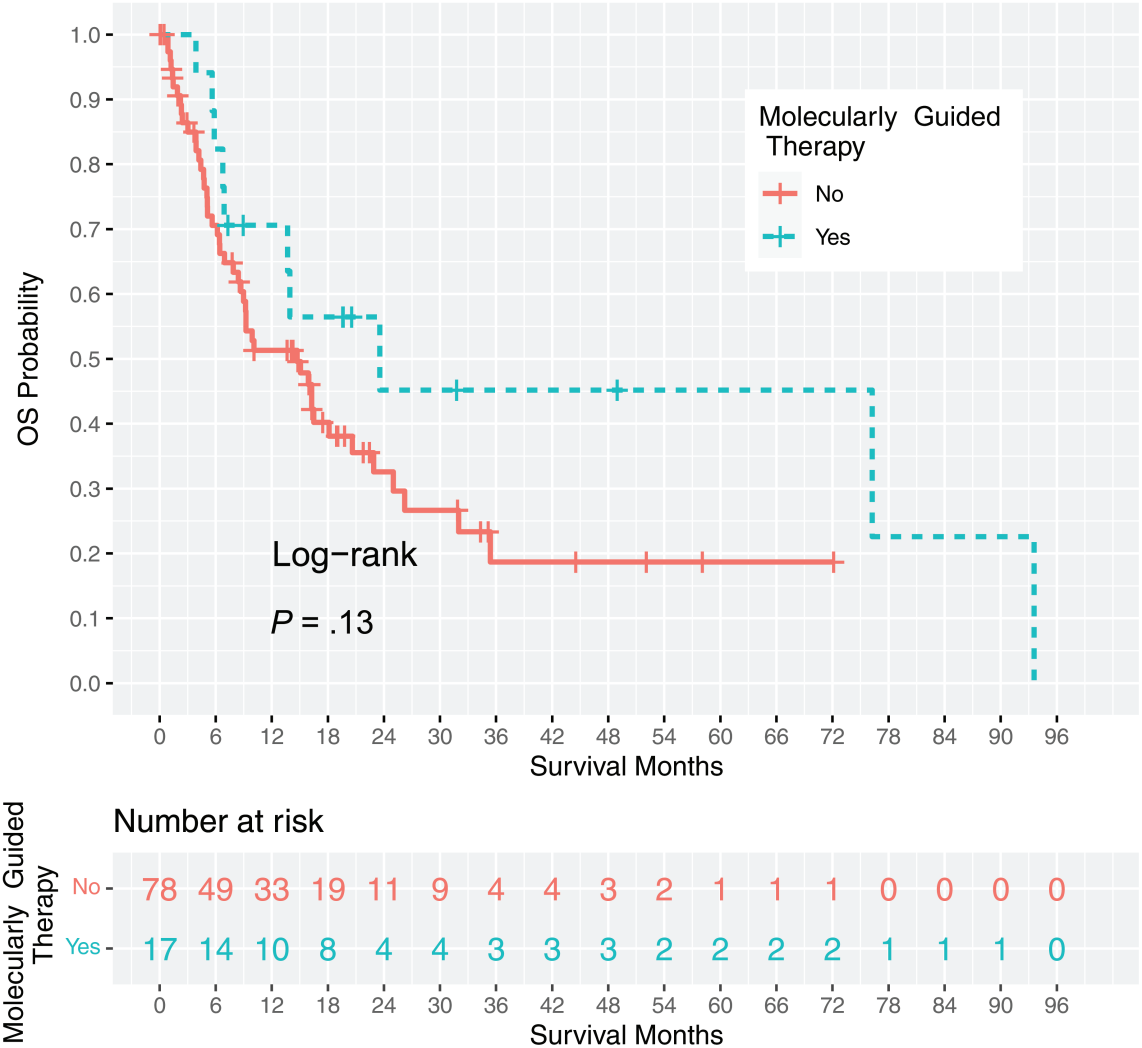
Overall survival (OS) Kaplan–Meier curves by therapy (molecularly guided vs standard options).

Thirty-three percent (*n* = 17) of patients with clinically actionable genetic alterations identified by NGS testing received molecularly guided therapy. Median overall survival was longer among patients treated with molecularly guided therapy (*n* = 17) compared with patients treated with standard options (*n* = 78), although this observation did not reach statistical significance (23.6 months vs 14.7 months; hazard ratio for death, 0.568; 95% confidence interval [CI], 0.268 to 1.205; *P* = .13; Figure 2).

**Figure 3.**
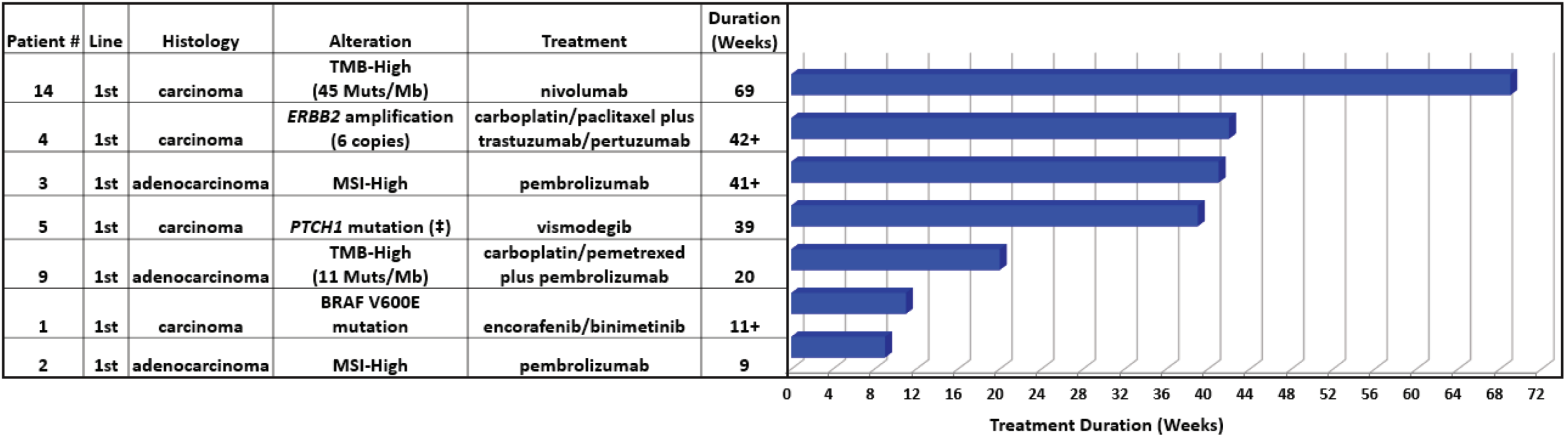
Duration of molecularly guided therapy for patients treated in the first line of therapy. (Mutation predicted to cause a loss-of-protein function (‡).).

**Figure 4.**
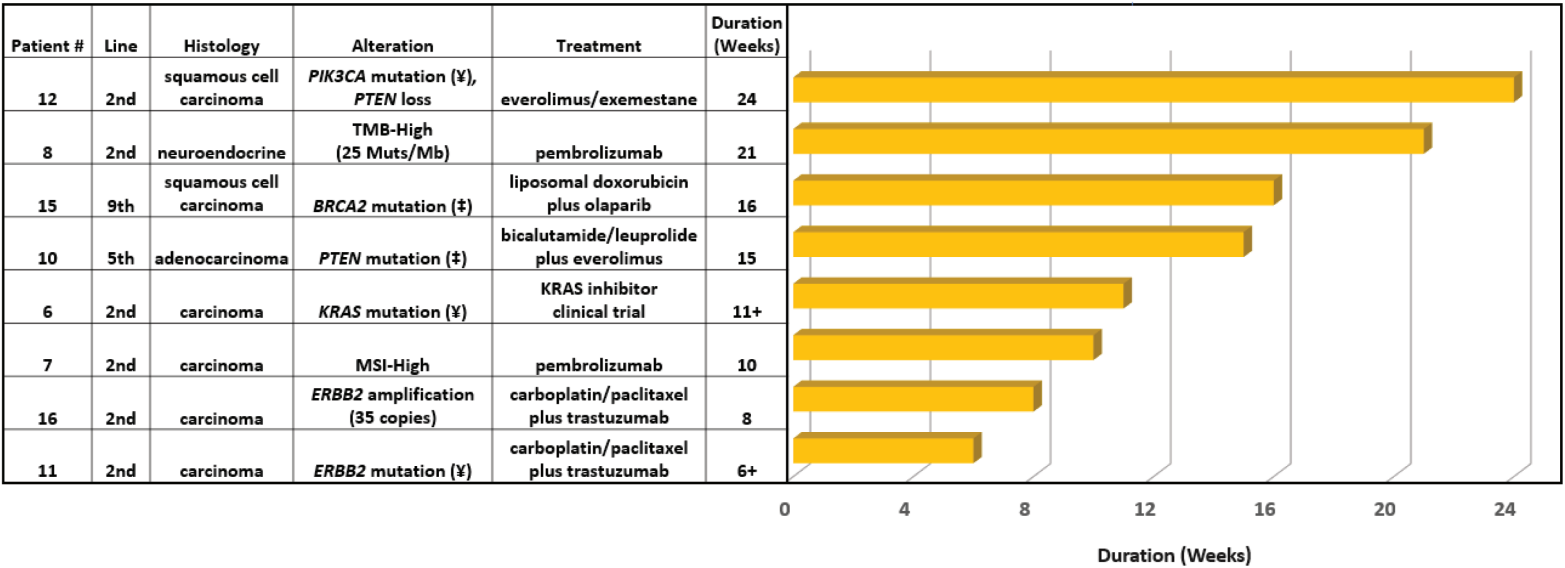
Duration of molecularly guided therapy for patients treated in the second line or later. (Loss-of-function mutation (‡), activating mutation (¥)).

Fifteen patients were evaluable for the duration of treatment. Of the 15 evaluable patients, seven (47%) received molecularly guided therapy as a first-line treatment. The median treatment duration was 39 weeks (range: 9 to 69 weeks; Figure 3). Among the 8 (53%) patients receiving molecularly guided therapy in the second- or later-line setting, the median duration of treatment was 13 weeks (range: 6 to 24 weeks; Figure 4). The Von Hoff ratio (PFS2/PFS1) was also determined for the patients receiving molecularly guided therapy in the second- or later-line setting. Thirty-eight percent (3 of 8) had a molecularly guided treatment with a duration that was ≥1.3 times the prior line of therapy (Supplementary Figure S1). An additional three cases (38%) received molecularly guided treatment for as long or longer than their prior line of treatment, but did not exceed the prespecified ratio of ≥1.3 as a surrogate marker of clinical benefit (Supplementary Figure S1). For all patients, inclusive of any line of therapy, molecularly guided treatment with targeted therapy or immunotherapy resulted in median treatment durations of 15 weeks (range, 6+ to 42 weeks) and 21 weeks (range: 9 to 69 weeks), respectively.

**Figure 5.**
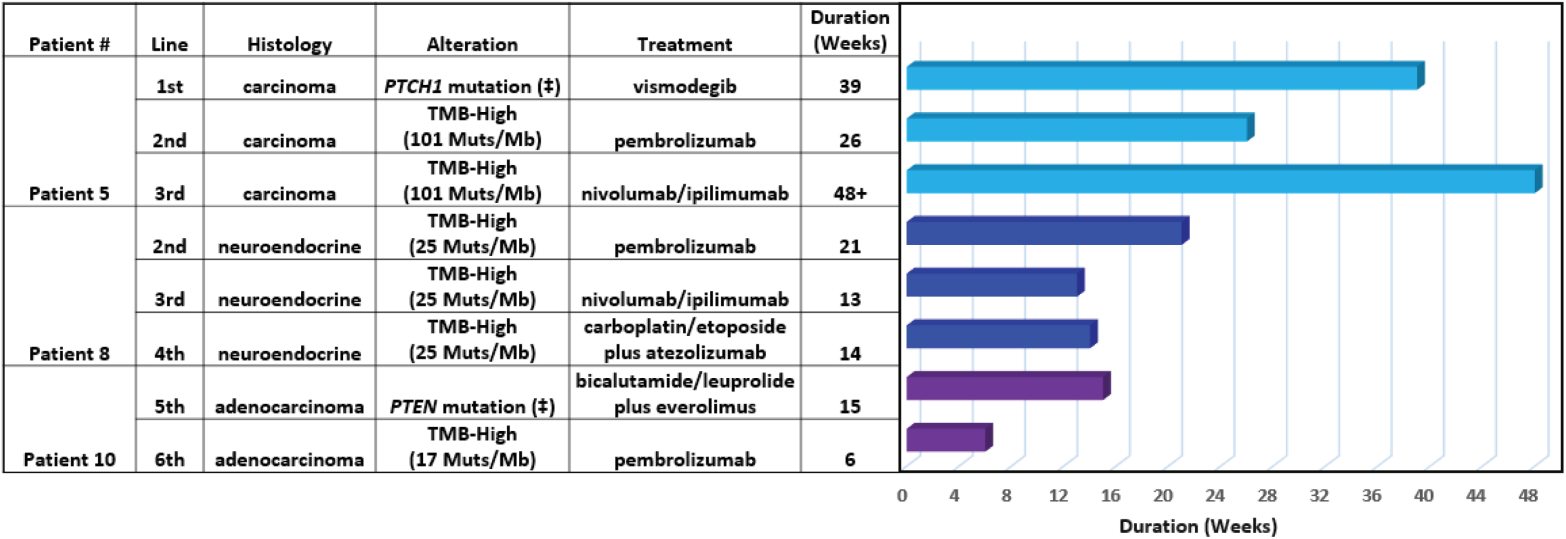
Treatment durations for three cases that received molecularly guided treatments for multiple lines of therapy. (Loss-of-function mutation (‡), activating mutation (¥)).

Three patients received molecularly guided treatment for multiple lines of therapy. The cumulative duration of molecularly guided therapy for these cases ranged from 21 to 113 weeks (Figure 5). This included two cases treated with both targeted and immunotherapy options as well as the identification of a patient with a high tumor mutational burden (25 Muts/Mb) who was subsequently treated with multiple lines of immunotherapy.

### Diagnosis

In our cohort, NGS assisted with diagnosis for 15% (*n* = 14) of cases. In the majority of these cases (13 of 14), a differential diagnosis had been established with two or three potential diagnoses possible (Supplementary Appendix 1). Examples of tumor types where the molecular features provided additional information include cholangiocarcinoma (*IDH1* R132C and *BAP1* splice site), colorectal cancer (MSI-High with *BRAF* V600E mutation), and non-small-cell lung cancer (*KRAS* G12D and *STK11* frameshift mutation). In all three of these examples, genomics alone would not have been sufficient to render a diagnosis but taken in the context of clinical features, imaging, and pathology review, a diagnosis was established.

### Pathogenic Germline Mutations

Four patients were referred to the Genetic Risk Assessment Service and had germline genetic testing performed based upon somatic test findings. After germline testing was performed, 75% (3 of 4) of cases were found to have pathogenic germline mutations involving *ATM*, *BRCA1*, or *CHEK2* genes.

## Discussion

Our study sought to describe the clinical utility of NGS test results to guide treatment decisions in a cohort of patients with CUP. Additional therapeutic options were identified for more than half of patients undergoing NGS. The median overall survival for CUP patients treated with molecularly guided treatment was 23.6 months. The reported hazard ratio of 0.568 amounts to a risk reduction of 43.2% of death in the molecularly guided treatment group compared with the standard group. We hypothesize that the reason why the comparison of overall survival in the molecularly guided versus standard options did not produce significance at the 0.05 alpha level is because the data set is both small and suffers from imbalance (with fewer patients in the molecularly guided treatment group than the standard options group) which is common in observational studies. To the best of our knowledge, this is the first study to provide survival data for CUP patients treated with a molecularly guided treatment approach. As the majority of CUP patients have a poor prognosis with overall survival of 3 to 10 months,^3-6^ this study demonstrates that molecularly guided treatment can provide meaningful clinical benefit. It has been reported that CUP patients treated at tertiary care centers may have a longer overall survival (~13 months) presumably due to patients being fit enough to travel for consultation at a tertiary care center.^25^ Our findings are consistent with that observation as the patients treated with standard options at our institution had a higher median overall survival (14.7 months) than what has been reported in the literature.^3-6^

Other surrogate measures of clinical benefit were also favorable in patients treated with molecularly guided therapy. The median duration of molecularly guided treatment in the first line of therapy was 39 weeks (range: 9 to 69 weeks). Among the Von Hoff ratio evaluable cases treated in the second line or later, 75% had a molecularly guided treatment duration that was as long or greater than that of their prior regimen with a median treatment duration of 13 weeks. Additionally, three patients received molecularly guided treatment for multiple lines of therapy, with cumulative durations of molecularly guided treatment ranging ~5 to ~28 months. It should be noted that one patient who received molecularly guided treatment was treated with everolimus based upon an identified *PTEN* loss-of-function mutation (level 4 evidence) (Figure 4).

In 2017, Varghese et al utilized the OncoKB classification to demonstrate that ~30% of CUP cases harbored clinically actionable biomarkers with a level 3 of evidence or greater.^25^ Compared with the study by Varghese et al,^25^ the present study identified a greater proportion of cases with clinically actionable biomarkers (55%) with level 3 or greater evidence. This finding can be attributed to the rapidly expanding armamentarium of drugs targeting specific genomic alterations or signatures. Since the publication by Varghese et al,^25^ there have been more than 25 biomarker-based oncology drug approvals for solid-tumor malignancies based upon an identified genomic alteration or signature predictive of drug response.^26^ Notably, tumor mutational burden has now been added to the list of tumor-type agnostic FDA approvals.^12,19^ The identification of a subset of CUP patients with a high tumor mutational burden suggests that there is a subgroup of CUP patients that may stand to benefit from immunotherapy options. The CUP patients in the present cohort treated with immune checkpoint inhibition had a median treatment duration of ~5 months, which is comparable to the treatment duration reported in a tumor-type agnostic clinical trial of pembrolizumab for TMB-High cancers, which reported a median treatment duration of 4.9 months.^27^

It is worth noting that in the early years of this retrospective evaluation TMB was not well established as a biomarker for immune checkpoint inhibition and this may have affected the number of patients receiving treatment based upon this biomarker in the present study. Prospective studies assessing the response to immune checkpoint inhibition in TMB-High CUP are warranted.

Alterations predicted to affect homologous recombination were a recurrent feature in our cohort suggesting the potential for utilization of poly (ADP-ribose) polymerase inhibitors (PARPi) or other therapeutics that target the DNA damage response pathway in select CUP patients.^28^ One illustrative patient case in our cohort describes a male patient with a pathogenic *BRCA2* alteration who received olaparib (PARPi) in combination with liposomal doxorubicin as he was declining clinically. He remained clinically stable on therapy for 16 weeks after nine prior lines of therapy. This was 5.3 times longer than his prior line of therapy with docetaxel that lasted for only 3 weeks.

The efficacy of DNA cross-linking platinum–based chemotherapy is augmented in tumors demonstrating a homologous recombination repair-deficient phenotype.^29^ Consequently, platinum sensitivity has served as a surrogate marker of sensitivity to subsequent PARP inhibition in several tumor types.^30,31^ As many of the chemotherapy regimens utilized for the empiric treatment of CUP contain a platinum agent, platinum sensitivity may also serve as a clinically useful biomarker for helping to identify which patients with CUP harboring mutations in DNA damage response genes may benefit from PARP inhibition.

*KRAS* mutations were another recurrent feature in our cohort which is consistent with prior reports describing the genomic landscape of CUP.^32,33^ In the present study, *KRAS* G12C mutations were identified in 7% (7 of 95) of CUP cases. Covalent inhibitors of *KRAS* G12C have demonstrated response rates of 32% to 45% in NSCLC with activity observed in other tumor types as well (eg, pancreatic, cholangiocarcinoma, endometrial, and ovarian cancers).^34,35^ Recently, the *KRAS* G12C inhibitor sotorasib was approved for locally advanced or metastatic *KRAS* G12C-mutated non-small-cell lung cancer patients who have received at least one prior systemic therapy. Studies are ongoing to evaluate combinations of *KRAS* G12C inhibitors with other agents (eg, anti-EGFR mAb or SHP2 inhibition).^36,37^ As novel therapeutics targeting KRAS emerge, this may provide additional targeted therapy options for those diagnosed with a CUP.

The finding that somatic NGS was able to identify patients for genetic counseling and subsequent germline testing should not be overlooked as three of the four patients with somatic NGS results suspicious for germline variants were confirmed to have inherited cancer susceptibility. Our results support prior studies describing the therapeutic and prognostic importance of germline testing.^38,39^ The criteria for referral to genetic counseling based on somatic NGS results are evolving, and may take into consideration the gene (eg, *BRCA1* or *BRCA2*), mutation allele frequency (eg, allele frequency *>* 50%) or a combination of both. If NGS results and clinicopathologic features are predictive of certain cancer types such as pancreatic or ovarian, consideration should also be given to genetic counseling referral. In addition to identifying targetable genetic alterations, cascade genetic testing of family members could allow for preventative screening where applicable.

### Molecular Features Assisting with Diagnosis

The concept that molecular features may assist with clinicopathologic diagnosis has previously been demonstrated, with Tothill et al showing that NGS results identified mutational profiles specific to certain subsets of malignancies in 69% of unknown primary cancers.^20^ Due to efforts by the Cancer Genome Atlas consortium and others, the genomic landscape has been described for numerous cancers with certain mutations or combinations of mutations occurring at a higher incidence in a particular cancer type.^40,41^ While targeted NGS is not yet intended to provide a diagnosis, it does provide additional information that may support a likely primary tumor type in the appropriate clinical and pathologic context. In our study, much as observed by Tothill et al, NGS was of the greatest utility when incorporated in the context of a full work-up with clinical features, imaging, and pathology review. Incorporating NGS results with clinicopathologic features to render a diagnosis may have treatment implications including the selection of a chemotherapy regimen, though the clinical benefit of utilizing molecular signatures to tailor therapy has not been fully elucidated. Clinical trials assessing gene expression profiling in those diagnosed with a CUP to predict tumor origin did not show a difference in PFS or overall survival between site-specific therapy versus empirical chemotherapy.^42,43^ However, these trials were enriched for metastatic pancreatic cancer which is known to have a poor response to therapy. Furthermore, the gene expression assays utilized were unlikely to identify those with favorable features for checkpoint immunotherapy including MSI or a TMB-High.

### Limitations

A limitation to this study is the use of several different NGS assays in the course of clinical care. Liquid biopsy with Guardant360 only interrogated ~74 genes (dependent upon the version of the assay), while the tissue-based assays such as FoundationOne CDx interrogated up to 324 genes. It is therefore not feasible to draw conclusions about any one assay, but does allow for more broad conclusions to be drawn regarding the clinical utility of NGS that may be readily applied in “real-world” clinical practice where a variety of NGS assays may be ordered in the course of clinical care. A second limitation is that, in our survival analysis, patients were not stratified by gender, age, performance status, comorbidities, or metastatic sites of disease, all of which can have prognostic implications.^1,3,5,44^ This study was performed as a single-institution retrospective, non-randomized analysis and progression-free survival per RECIST 1.1 criteria could not be determined. For those patients with a molecularly guided option identified, the reason for selecting or not selecting a targeted therapy as a line of treatment could not be determined. Additionally, CUP patients undergoing NGS over a 5-year period were included in this study. Decision-making for treating CUP patients with targeted therapy or checkpoint immunotherapy likely evolved over time as additional evidence emerged, which could have influenced outcomes. Prospective studies are warranted to further evaluate the clinical benefit of NGS for patients diagnosed with CUP and the ongoing CUPISCO study (NCT03498521) will offer additional data in the future.^33,45^

## Conclusion

Our retrospective analysis demonstrates that CUP patients may derive clinical benefit from molecularly guided treatment approaches much as a patient would with a known primary tumor type.^46^ In our single-institution cohort, the median overall survival of 23.6 months for CUP patients treated with molecularly guided therapy compares favorably to historical data suggesting a need for further prospective investigation. Our data also identified a higher percentage of patients eligible for molecularly guided therapy compared with prior reports, plausibly due to increasing treatment options with molecularly guided indications and histology agnostic indications for immunotherapy. NGS also demonstrated clinical utility in assisting with diagnosis in a small subset of patients based upon identified molecular features and also helped to identify a small group of patients likely to harbor pathogenic germline mutations. Our findings support the recommendations of the ESMO Precision Medicine Working Group and National Comprehensive Cancer Network for the use of NGS in the management of patients with cancer of unknown primary with close collaboration between precision medicine specialists, pathologists, radiologists, and medical oncologists.

## Supplementary Material

oyab014_suppl_Supplementary_Appendix_1

oyab014_suppl_Supplementary_Figure

## Data Availability

The data underlying this article will be shared at reasonable request to the corresponding author.
