# Supplementary material for: Evaluation of Targeted Next-Generation Sequencing for the Management of Patients Diagnosed with a Cancer of Unknown Primary
Source: Oncologist. 2022 Jan 28;27(1):e9–e17. doi: 10.1093/oncolo/oyab014 (PMC8842368; doi:10.1093/oncolo/oyab014)
Supplement: oyab014_suppl_Supplementary_Figure [file oyab014_suppl_supplementary_figure.pdf]

The Oncologist
The official journal of the Society for Translational Oncology

Supplemental Figure for:
Evaluation of Targeted Next-Generation Sequencing for the Management of Patients Diagnosed with a Cancer of Unknown Primary
J. Kevin Hicks et al.

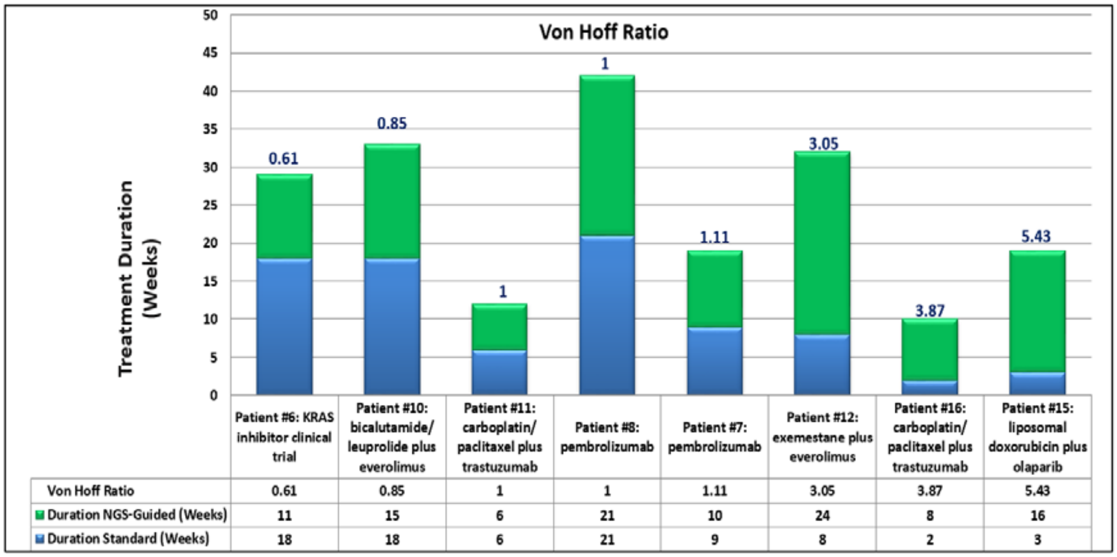

	Patient #6: KRAS inhibitor clinical trial	Patient #10: bicalutamide/ leuprolide plus everolimus	Patient #11: carboplatin/ paclitaxel plus trastuzumab	Patient #8: pembrolizumab	Patient #7: pembrolizumab	Patient #12: exemestane plus everolimus	Patient #16: carboplatin/ paclitaxel plus trastuzumab	Patient #15: liposomal doxorubicin plus olaparib
Von Hoff Ratio	0.61	0.85	1	1	1.11	3.05	3.87	5.43
Duration NGS-Guided (Weeks)	11	15	6	21	10	24	8	16
Duration Standard (Weeks)	18	18	6	21	9	8	2	3

Figure S1: The Von Hoff ratio (PFS2/PFS1) for the eight Von Hoff evaluable cases.
